# Upregulation of CCR4 in activated CD8^+^ T cells indicates enhanced lung homing in patients with severe acute SARS‐CoV‐2 infection

**DOI:** 10.1002/eji.202049135

**Published:** 2021-04-19

**Authors:** Silvia Spoerl, Anita N. Kremer, Michael Aigner, Nina Eisenhauer, Pauline Koch, Lina Meretuk, Patrick Löffler, Matthias Tenbusch, Clara Maier, Klaus Überla, Lucie Heinzerling, Benjamin Frey, Gloria Lutzny‐Geier, Thomas H. Winkler, Gerhard Krönke, Marcel Vetter, Heiko Bruns, Markus F. Neurath, Andreas Mackensen, Andreas E. Kremer, Simon Völkl

**Affiliations:** ^1^ Department of Internal Medicine 5, Hematology, and Oncology University Hospital Erlangen and Friedrich‐Alexander‐University Erlangen‐Nürnberg Erlangen Germany; ^2^ Institute of Virology University Hospital Erlangen and Friedrich‐Alexander‐University Erlangen‐Nürnberg Erlangen Germany; ^3^ Department of Dermatology University Hospital Erlangen and Friedrich‐Alexander‐University Erlangen‐Nürnberg Erlangen Germany; ^4^ Department of Radiation Oncology University Hospital Erlangen and Friedrich‐Alexander‐University Erlangen‐Nürnberg Erlangen Germany; ^5^ Division of Genetics, Department of Biology, Nikolaus‐Fiebiger‐Center for Molecular Medicine Friedrich‐Alexander‐University Erlangen‐Nürnberg Erlangen Germany; ^6^ Department of Internal Medicine 3, Rheumatology, and Immunology University Hospital Erlangen and Friedrich‐Alexander‐University Erlangen‐Nürnberg Erlangen Germany; ^7^ Department of Internal Medicine 1, Gastroenterology, Pneumology, and Endocrinology University Hospital Erlangen and Friedrich‐Alexander‐University of Erlangen‐Nürnberg Erlangen Germany

**Keywords:** CCR4, COVID‐19, Lung homing, SARS‐CoV‐2, T‐cell activation

## Abstract

COVID‐19 is a life‐threatening disease leading to bilateral pneumonia and respiratory failure. The underlying reasons why a smaller percentage of patients present with severe pulmonary symptoms whereas the majority is only mildly affected are to date not well understood.

Comparing the immunological phenotype in healthy donors and patients with mild versus severe COVID‐19 shows that in COVID‐19 patients, NK‐/B‐cell activation and proliferation are enhanced independent of severity. As an important precondition for effective antibody responses, T‐follicular helper cells and antibody secreting cells are increased both in patients with mild and severe SARS‐CoV‐2 infection. Beyond this, T cells in COVID‐19 patients exhibit a stronger activation profile with differentiation toward effector cell phenotypes. Importantly, when looking at the rates of pulmonary complications in COVID‐19 patients, the chemokine receptor CCR4 is higher expressed by both CD4 and CD8 T cells of patients with severe COVID‐19. This raises the hypothesis that CCR4 upregulation on T cells in the pathogenesis of COVID‐19 promotes stronger T‐cell attraction to the lungs leading to increased immune activation with presumably higher pulmonary toxicity.

Our study contributes significantly to the understanding of the immunological changes during COVID‐19, as new therapeutic agents, preferentially targeting the immune system, are highly warranted.

## Graphical abstract

Patients with mild COVID‐19 after SARS‐CoV2 infection exhibit increased T‐cell activation and induction of T‐cell exhaustion.

During severe COVID‐19 patients show massive T‐cell activation and upregulation of homing receptors promoting hyperinflammation and increased lung trafficking.

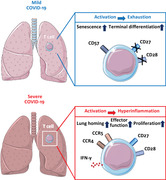

## Introduction

Coronavirus disease 2019 (COVID‐19) is an infection with SARS‐CoV‐2 (severe acute respiratory syndrome coronavirus 2) primarily affecting the respiratory tract, which initially emerged in December 2019. Within a few weeks, the number of infected cases rose rapidly and expanded to Europe [1]. In March 11, 2020, the WHO declared the pandemic.

The clinical presentation of the patients varies: most patients with COVID‐19 present with only mild to moderate symptoms such as cough, headache, and fever. However, a smaller percentage of patients, mainly those being older and bearing one or multiple comorbidities experience a more severe course of disease with pneumonia often leading to respiratory insufficiency and intensive care unit (ICU) treatment [2]. In those severe cases, lymphocytes are reported to be decreased and laboratory results reveal hypalbuminemia, elevated values of lactatehydrogenase, C‐reactive protein (CRP), and ferritin [3].

Causal treatment standards for severe COVID‐19 are still lacking, however, experimental treatment strategies are currently being studied including remdesivir [4], dexamethasone, or tocilizumab [5].

Our adaptive immune system plays a major role in fighting viral infections. Especially B cells, which produce neutralizing antibodies, but also CD4^+^ Th and CD8^+^ cytotoxic T cells are essential effector cells in antiviral responses. For the epidemic being caused by SARS‐CoV‐1 in 2002, it has been shown that the humoral immunity following the disease was protective, however, not enduring. In contrast, memory T cells persisted up to 6 years after the infection [6].

Cytotoxic lymphocytes, such as cytotoxic T lymphocytes (CTLs) and natural killer (NK) cells, are essential for controlling viral infections, and the functional exhaustion of cytotoxic lymphocytes could be correlated with disease progression [7]. Interestingly, COVID‐19 patients, especially those with severe infection, showed increased levels of inhibitory receptors, regulatory molecules, and decreased levels of multiple cytokines in peripheral blood T cells [8]. Different studies reported on decreased lymphocytes, especially CD8^+^ T lymphocytes, mainly in those patients being critically ill [9, 10, 11]. The reduced lymphocyte counts were often associated with exhausted phenotypes, characterized by specific markers as programmed cell death protein (PD)‐1 [12]. However, the role of immunomodulatory molecules and cellular mechanisms in SARS‐CoV‐2 infection and severity is not adequately understood. Beyond cytotoxic T cells, also T‐follicular helper cells (T_FH_), antibody secreting cells (plasmablasts) [13], and B cells [14] changed throughout the course of a SARS‐CoV‐2 infection [13].

As the adaptive immune system is crucial for a “COVID‐19‐specific” immune response and SARS‐CoV‐2 reactive CD4 T cells can be found throughout the course of an infection [15], we aimed to get closer insight into the lymphocyte profile of patients with COVID‐19 infection and how lymphocyte activation, differentiation, and trafficking differs depending on the severity of the disease.

## Results

### Patient characteristics

The major aim of our study was to determine the effect of SARS‐CoV‐2 infection on different immune cell subsets of lymphatic origin dependent on the severity of the disease. Previous studies have shown increased T‐cell activation in patients with active SARS‐CoV‐2 infection, however, it is not quite understood how COVID‐19 impacts on distinct lymphocyte populations, especially when it comes to comparing severe with mild cases of COVID‐19 disease.

For our study, we analyzed healthy donors and patients with mild (NIH Score 4 + 5) and severe (NIH Score 1‐3) cases of SARS‐CoV‐2 infection. Healthy donors had a median age of 35 years (range: 26‐46). In our control population, eight healthy donors were females and seven males. Patients’ characteristics are shown in Supporting information Table S1. All patients were screened positive for SARS‐CoV‐2 infection by PCR from a nose‐throat swab (Institute of Virology, University Hospital of Erlangen). Viral load at the time of blood draw already became negative in four of the patients with mild and in four of the patients with severe disease. Fifteen patients had a mild course of disease with a median age of 65 years (range: 18‐96). Median time after onset of disease symptoms until the day of sample collection was 5 days. Patients with mild courses presented with COVID‐19 specific, mostly respiratory symptoms, such as fever, dry cough, and shortness of breath (one patient was without symptoms), however, no one required treatment in the ICU, mechanical ventilation, nor died due to COVID‐19. A sum of 14 out of 15 patients (median age 69 years, range 37‐88) with a severe course of disease were treated in the ICU department of our hospital, all of them needed supplementary oxygen, with 11 of them requiring mechanical ventilation, and 3 of them were transferred to an extracorporeal membrane oxygenation support system. The remaining patient refused invasive ventilation or ICU treatment. Mortality rate of the cohort with severely ill patients was 33%. In severely diseased patients, blood samples were collected at a median of 11 days after the onset of disease symptoms. Since a previous study has discussed the effects of ACE inhibition in COVID‐19 patients [16], we compared the patients regarding their medication history of ACE inhibitors. We found that in the group of patients with a severe course of disease, 36% had taken ACE inhibitors when acquiring their SARS‐CoV‐2 infection compared to only 13% in the patients with mild course of disease (*p* = 0.16).

CRP, ferritin, and IL‐6 levels were significantly higher in the group with a severe disease course compared to patients only having mild symptoms (Supporting information Fig. S1).

### NK cells and B cells exhibit increased proliferation in COVID‐19 patients

**Figure 1:**
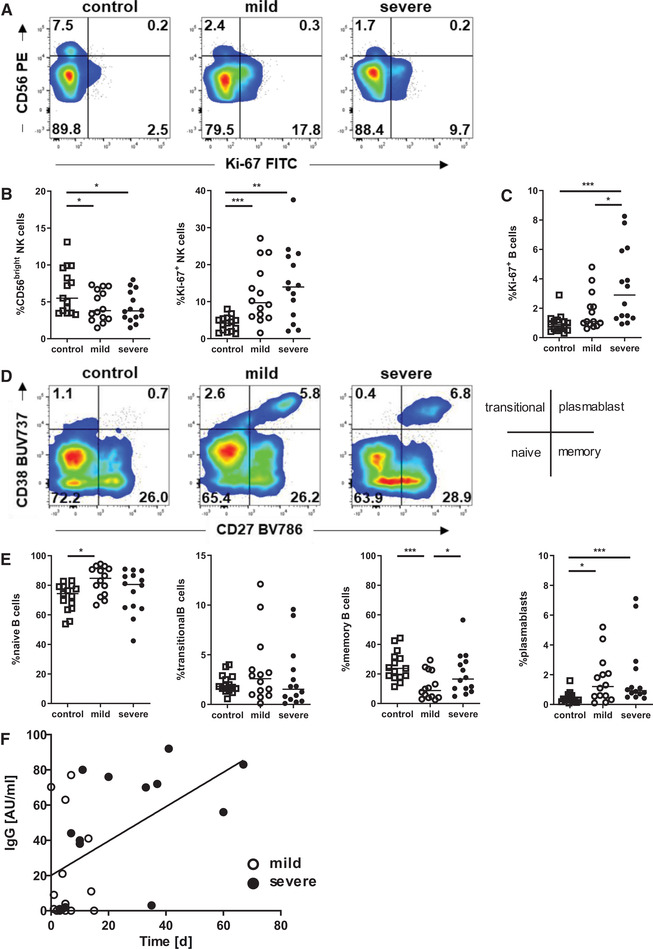
NK cells and B cells exhibit increased proliferation in COVID‐19 patients. (A, B) Samples from healthy controls and patients with mild and severe COVID‐19 infection were gated for NK cells and analyzed for CD56 and Ki‐67 expression by flow cytometry. (A) Density plots show a representative experiment out of 5 experiments with 6‐10 donors per experiment, (B) graphs exhibit cumulative data of all COVID‐19 patients (mild: open circle, n = 14; severe: black circle, n = 14) and healthy controls (open squares, n = 15). (C) Proliferation of B cells was analyzed by Ki‐67 staining, each symbol represent an individual subject (healthy controls: n = 15; mild: n = 14; severe: n = 14). (D) Expression of CD27 and CD38 were determined in B cells, density plots show a representative experiment out of 5 experiments with 6‐10 donors per experiment. Diagram defines B‐cell subsets and data were measured by flow cytometry. (E) Frequencies of naïve B cells (CD27^–^/CD38^–^), transitional B cells (CD27^–^/CD38^+^), memory B cells (CD27^+^/CD38^–^), and plasmablasts (CD27^high^/CD38^high^) among all live B cells are shown. Healthy controls (n = 15) and patients with mild (n = 14) and severe COVID‐19 infection (n = 14) are compared. (B, C, E) Bars represent median, significance within these cohorts is calculated using Mann–Whitney‐U test with *<0.05, **<0.01, and ***<0.001. (F) SARS‐CoV‐2‐specific IgG titers in patients with mild (white dots) and severe (black dots) COVID‐19 are depicted as measured on the indicated day after onset of symptoms by chemiluminescent Immunoassay (CLIA**)**. Data from 2 experiments with 10‐20 donors per experiment *r* = 0.5272, **p < 0.01 (Spearman's test).

Given that lymphocyte numbers and composition have been associated with disease severity and progression [17, 18], we studied the lymphoid compartment in COVID‐19 patients using high‐dimensional flow cytometry (Supporting information Fig. S2). First, we analyzed the NK cell phenotype and found a low frequency of CD56^bright^ NK cells and enhanced expression of the proliferation marker Ki‐67 in patients with active COVID‐19 infection compared to healthy individuals (Fig. 1A and B). Moreover, CD56^dim^ NK cells of patients with severe disease tended to exhibit low CD16 coexpression (gating strategy is depicted in Supporting information Fig. S2), a phenotype associated with increased cytotoxicity [19], whereas NKG2A expression was heterogeneously expressed in all analyzed samples (Supporting information Fig. S3A and B). Analysis of the B‐cell compartment also demonstrated increased proliferation in severely diseased COVID‐19 patients (Fig. 1C). Subsets of naïve and transitional B cells were largely comparable in all individuals, while memory B cells were reduced in patients with mild disease (Fig. 1D and E). Antibody production is crucial in establishing enduring immunity, in this context we looked at plasmablasts determined by their expression of CD38^high^/CD27^high^. Interestingly, plasmablasts were increased in patients with mild and severe COVID‐19 disease, indicating that antibody responses were not only initiated in mildly diseased patients, but were also maintained in severe courses of the disease. Moreover, the observed B‐cell proliferation was due to an increase in plasmablast frequency, as this subset highly expresses Ki‐67 (data not shown). This was in line with robust antibody titers in both groups (Fig. 1F), with later time points in the duration of disease leading to higher antibody titers.

### COVID‐19 patients with severe disease show enhanced T‐cell activation and differentiation

**Figure 2:**
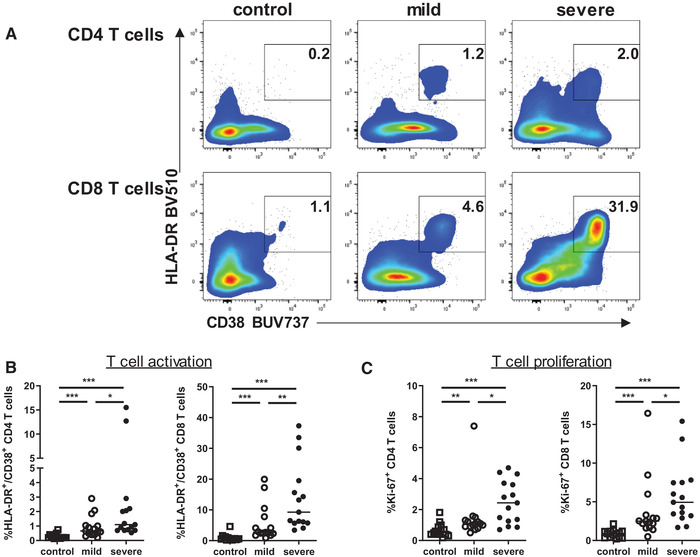
CD4 and CD8 T cells exhibit increased activation in patients with severe COVID‐19 infections. (A) Activation of CD4 and CD8 T cells was determined by CD38 and HLA‐DR expression using specific antibodies and measured by flow cytometry. Density plots show representative experiments out of 5 experiments with 6‐10 donors per experiment. (B, C) Graphs display percentages of activated (HLA‐DR^+^/CD38^+^) (B) and proliferating (C) cells within CD4 and CD8 T‐cell subsets in healthy controls (n = 15, open square) and patients with mild (n = 15, open circle) and severe (n = 15, black circle) COVID‐19 disease. Bars represent median. *<0.05, **<0.01, and ***<0.001 (Mann–Whitney U test).

T‐cell activation, proliferation, and effector cell differentiation is essential in forming an effective T‐cell mediated antiviral immune response. We first analyzed activation of CD4 Th cells and cytotoxic CD8 T cells by HLA‐DR and CD38 expression [20] and found a clear increase in the activation of both T‐cell subsets in patients with COVID‐19 disease (Fig. 2A and B). Interestingly, activation levels were further increased in patients with severe courses of COVID‐19 disease. Consistent with this finding, CD4 and CD8 T‐cell proliferation was also enhanced in COVID‐19 patients, with further increase in severe cases (Fig. 2C). Of note, the CD8 T‐cell population showed much higher activation and Ki‐67 expression than the CD4 T cells, suggesting its prominent role in response to viral infection.

**Figure 3:**
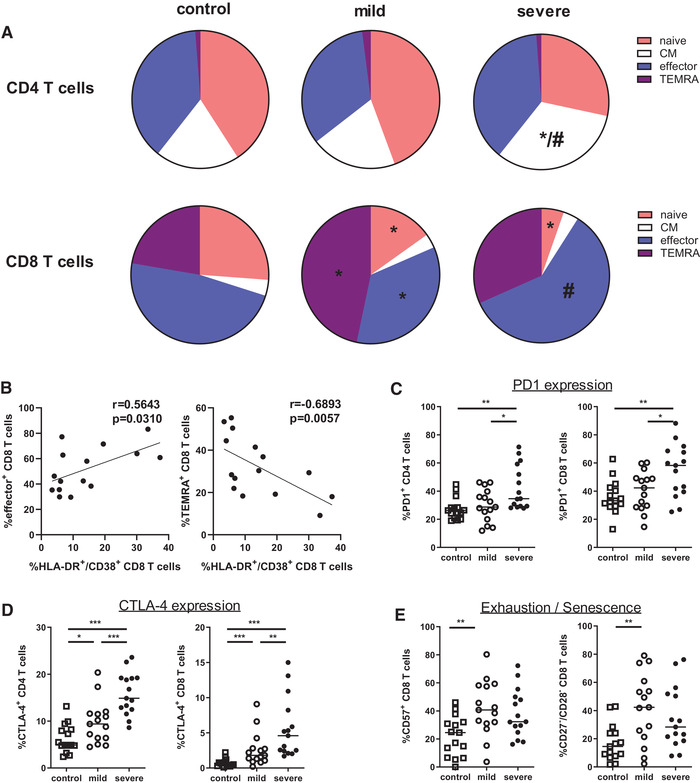
SARS‐CoV‐2 infection induces enhanced T‐cell differentiation. (A) Naïve (CCR7^+^/CD45RO^–^), central memory (CM, CCR7^+^/CD45RO^+^), effector (CCR7^–^/CD45RO^+^), and TEMRA (CCR7^–^/CD45RO^–^) CD4 and CD8 T cells were determined by flow cytometry. Pie charts summarizing the frequencies of naïve, CM, effector, and TEMRA cells within CD4 and CD8 T cells of healthy controls (n = 15) and patients with mild and severe COVID‐19 infection (n = 15, respectively). Data are cumulative of five experiments. Statistical differences were tested using Mann–Whitney U test. Stars (*) represent mild and severe COVID‐19 patients compared to control (*p* <0.05), hashtag (#) represents mild versus severe disease (*p* < 0.05), respectively. (B) Correlation of percentages of HLA‐DR^+^/CD38^+^ cells and effector cells (left) or TEMRA cells (right) among all CD8 T cells from COVID‐19 patients with severe disease (n = 15) is shown. Spearman`s rank test was used for correlation analysis. (C, D) Graph shows percentage of PD1^+^ and CTLA‐4^+^ cells within CD4 T cells (C) and CD8 T cells (D) in healthy controls (n = 15, open square) and patients with mild (n = 15, open circle) and severe (n = 15, black circle) COVID‐19 disease. (E) CD8 T cells of healthy controls and patients with mild and severe COVID‐19 disease were analyzed for expression of CD57, CD27, and CD28. Cumulative data from five experiments of all healthy controls and COVID‐19 patients are graphed, each symbol represents an individual subject and measured by flow cytometry. Bars represent median, *<0.05, **<0.01, and ***<0.001 (Mann–Whitney U test).

We next examined the differentiation of CD4 T cells and found no significant differences between healthy individuals and COVID‐19 patients with mild course (Fig. 3A and Supporting information Fig. S4A and B). However, patients with severe disease showed an expansion of central memory (CM) CD4 T cells pointing to an active immune response. Notably, the CD8 T‐cell compartment in patients with mild COVID‐19 exhibited a shift toward terminally differentiated effector‐memory cells re‐expressing CD45RA (TEMRA), highly differentiated cells with effector functions but limited mitotic activity and survivability [21]. Moreover, frequencies of naïve and effector CD8 T cells decreased in these patients. The decline in the naïve CD8 compartment was further enhanced in patients with severe disease. Interestingly, severe cases showed an expansion of effector cells, while the induction of TEMRA cells could not be observed (Fig. 3A and Supporting information Fig. S4A and B). Furthermore, we found a significant correlation of CD8 T‐cell activation and differentiation in patients with severe SARS‐CoV2 infection as high activation state is associated with increased effector and decreased terminal differentiation (Fig. 3B).

In an attempt to understand if exhaustion or senescence could have an effect in regulating immune responses in COVID‐19 patients, we further analyzed CD4 and CD8 T cells for markers associated with cell regulation, exhaustion, and senescence. As previously reported, frequency of PD1^+^ cells was increased in COVID‐19 patients [22]. However, subdivision of COVID‐19 patients in mild and severe cases resulted in high expression of PD1 in CD4 and CD8 T cells of severe disease, whereas patients with mild SARS‐CoV‐2 infection were comparable to healthy individuals (Fig. 3C and Supporting information Fig. S5A). In addition, expression of the coinhibitory receptor CTLA‐4 was also upregulated in CD4 and CD8 T cells of patients with severe COVID‐19 (Fig. 3D). Given their role in antiviral immune response, we further analyzed CD8 T cells for additional markers of terminal differentiation and senescence. Confirming the induction of terminal differentiation in mild COVID‐19, expression of CD57 was increased in CD8 T cells compared to healthy individuals (Fig. 3E). Patients with severe disease also showed heightened CD57 expression, but they did not reach the level of mild cases. Moreover, we measured frequencies of senescent CD8 T cells and observed a profound expansion of CD27^–^/CD28^–^ cells in COVID‐19 patients with mild disease (Fig. 3E and Supporting information Fig. S5B). Notably, patients with severe disease did not show augmentation of senescent CD8 T cells. Due to the high heterogeneity of COVID‐19 patients, the difference in CD57 and CD27/CD28 expression levels did not reach statistical significance. However, the percentage of senescent CD27^–^/CD28^–^ CD8 T cells was inversely correlated with CD8 T‐cell activation level in patients with severe disease (Supporting information Fig. S5C). In summary, we found an increase in terminal differentiation and senescence in patients with mild symptoms, while severe disease was associated with upregulation of effector differentiation, indicating that severity of the disease might be due to a failure in the contraction phase of the immune response.

### SARS‐CoV‐2 infection modulates T‐cell functionality

**Figure 4:**
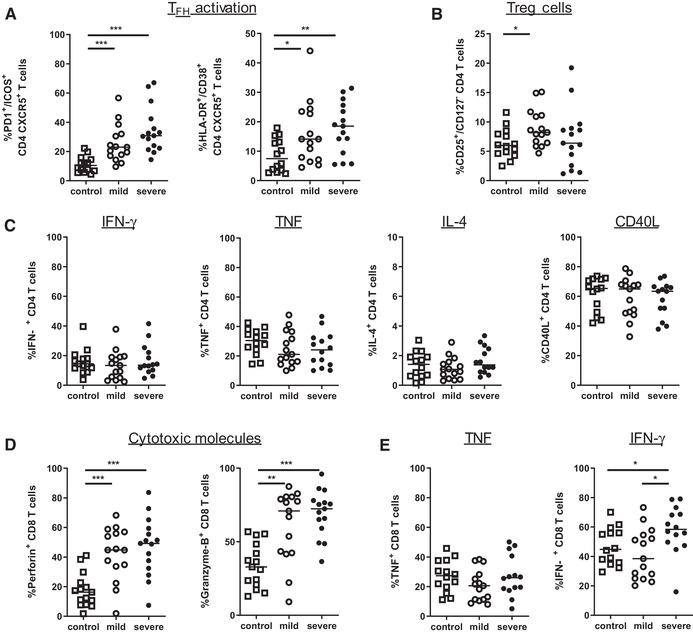
COVID‐19 influence T‐cell functionality. (A) Frequencies of circulating T_FH_ cells and activated (HLA‐DR^+^/CD38^+^) circulating T_FH_ cells were determined in healthy controls (n = 14), and patients with mild and severe COVID‐19 disease (n = 15, respectively). (B) Graph represents percentage of Treg cells among CD4 T cells in all three cohorts. (C) PBMC were stimulated with PMA/ionomycin in the presence of Golgi Stop. Expression of indicated cytokines and CD40L was determined in CD4 T cells by intracellular flow cytometry staining (each group n = 14). (D) CD8 T cells were analyzed for expression of the cytotoxic molecules Perforin and Granzyme‐B (each group n = 15). (E) Expression of IFN‐γ and TNF in CD8 T cells after stimulation with PMA/ionomycin in the presence of Golgi Stop (each group n = 14). Cumulative data from five experiments and each dot represents an individual healthy donor (control, open square), or COVID‐19 patient with mild (open circle) of severe disease (black circle). Data were measured by flow cytometry. Significance within these cohorts is calculated using Mann–Whitney U test with *<0.05, **<0.01, and ***<0.001.

CD4 Th cell properties are critically involved in providing help to B cells and supporting antibody production. A subpopulation of CD4 T cells, described as T_FH_, is characterized by CXCR5 expression and expresses activation markers such as ICOS or PD1. T_FH_ are typically increased in autoimmune and inflammatory responses and they were attributed a crucial role in HIV infection [23]. We found the PD1/ICOS double‐positive population of circulating CD4 CXCR5+ T_FH_ to be strongly upregulated in COVID‐19 patients (Fig. 4A, Supporting information Fig. S6). Furthermore, CD4 CXCR5+ T_FH_ were more activated in severe cases of disease. Additionally, we found an increase of CD4 CD127^–^ CD25^+^ T cells (Fig. 4B, Supporting information Fig. S6) in mildly diseased COVID‐19 patients. This population is consistent with FOXP3^+^ expressing Tregs according to previous publications by Koreth and colleagues [24].

As COVID‐19 severity might affect T‐cell functionality, we wanted to determine if the cytokine secretion of the respective T‐cell population was impaired. Expression of cytokines IFN‐γ, TNF, IL‐4, and the costimulatory molecule CD40L in CD4 T cells was comparable in healthy individuals and COVID‐19 patients, with some mildly diseased patients seeming to express lower amounts of TNF (Fig. 4C and Supporting information Fig. S7A). Consistent with increased cell differentiation in COVID‐19 patients, the cytotoxic molecules perforin and granzyme B were strongly upregulated in CD8 T cells (Fig. 4D and Supporting information Fig. S7B). Expression of TNF was not modulated in CD8 T cells from COVID‐19 patients, while IFN‐γ production was only increased in severe courses of disease (Fig. 4E). Systemic levels of proinflammatory cytokines tended to be in line with expression in T cells, with low and comparable levels for IFN‐γ, TNF, GM‐CSF, IL‐12/IL‐23p40, IL‐15, and IL‐17A in mild and severe cases (Supporting information Fig. S7C).

Taken together, COVID‐19 patients showed enhanced activation of T_FH_ and cytotoxic CD8 T cells, which tended to be associated with disease severity

### Altered chemokine receptor expression in severe COVID‐19 attracts T cells to the lungs

In immune activation, chemokine receptors are upregulated due to inflammatory responses and homing receptor expression is crucial for trafficking of the immune cells. T cells express specific chemokine and homing receptors supporting their migration to lymphoid tissues for antigen encounter or inflammatory regions in order to exert local immune responses.

**Figure 5:**
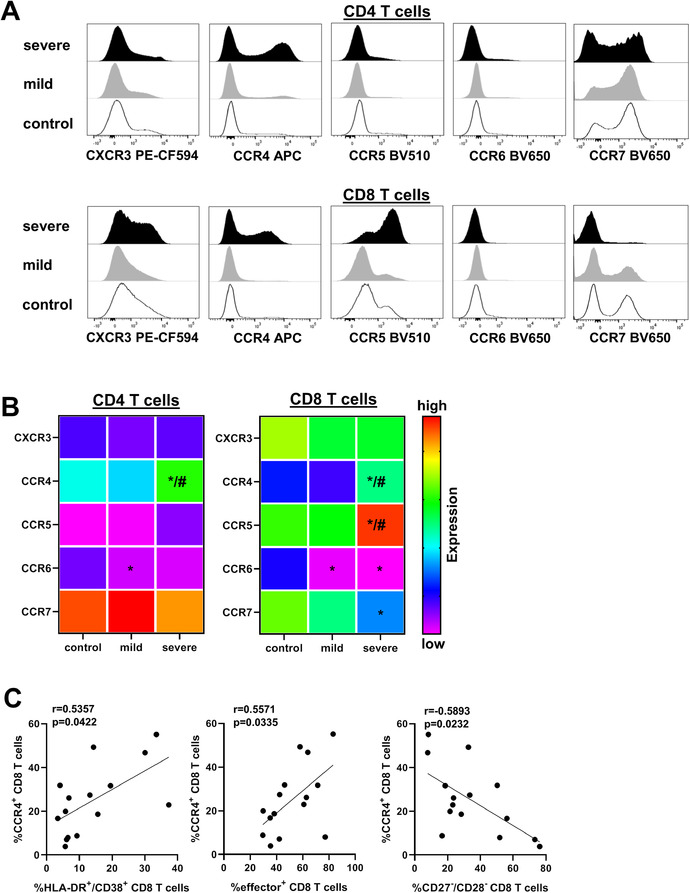
COVID‐19 patients with severe disease exhibit expression of proinflammatory and lung‐homing receptors. Expression of chemokine receptors CXCR3, CCR4, CCR5, CCR6, and CCR7 were determined by flow cytometry in CD4 and CD8 T cells of healthy controls and COVID‐19 patients. (A) Representative histograms depicting levels of receptor expression as mean fluorescence intensity (MFI) are shown, Y‐axis is normalized to mode. (B) Heat map summarizing the frequencies of CXCR3^+^, CCR4^+^, CCR5^+^, CCR6^+^, and CCR7^+^ cells within CD4 T cells (left) and CD8 T cells (right) of healthy controls (n = 15) and COVID‐19 patients with mild (n = 15) and severe (n = 15) disease is shown. Cumulative data from five experiments. Statistical differences were analyzed using Mann–Whitney U test. Stars (*) represent mild and severe COVID‐19 patients compared to control (*p* < 0.05), hashtag (#) represents mild versus severe disease (*p* < 0.05), respectively. (C) Correlation of percentages of CCR4^+^ and HLA‐DR^+^/CD38^+^ cells, effector cells, and CD27^–^/CD28^–^ cells among all CD8 T cells from COVID‐19 patients with severe disease (n = 15) is shown. Significance was calculated using Spearman's test.

Chemokine receptors are differentially expressed on T cells indicating their particular Th cell subset. In this context, CXCR3 is expressed mainly on proinflammatory Th1 cells, whereas CCR6 can be found predominantly on Th17 cells. Beyond that, CCR4 is associated with Th2 differentiation and contributes to T‐cell lung imprinting [25]. To determine whether T cells might be primed for homing to specific tissues during SARS‐CoV‐2 infection and whether chemokine receptors affect T‐cell subsets in mild and severe COVID‐19 cases, we checked for particular chemokine receptors in our cohorts. Strikingly, we found the lung‐homing receptor CCR4 and the proinflammatory receptor CCR5 strongly upregulated on CD8 T cells in patients with severe COVID‐19 infection, mainly characterized by pulmonary failure and the requirement of mechanical ventilation, however, not in mild COVID‐19 disease (Fig. 5A and B and Supporting information Fig. S8A and B). Moreover, CD8 T cells from patients with severe disease exhibited reduced CCR7 expression, pointing to enhanced attracting to sites of inflammation and limited homing to secondary lymphoid organs. Furthermore, CD4 T cells showed higher CCR4 expression, which was restricted again to severe cases. In contrast, CCR6 expression was downregulated in CD8 T cells of both mild and severe disease, whereas CXCR3 expression remained quite unchanged within the different cohorts. Interestingly, further analysis revealed a linear relationship between CCR4 expression and CD8 T‐cell activation and effector differentiation in patients with severe disease (Fig. 5C). In addition, increased CCR4 expression was inversely correlated with induction of senescent CD8 T cells. We also observed significantly increased levels of ICAM‐1 in patients with severe course of disease as compared to those with mild symptoms (Supporting information Fig. S8C).

In summary, CCR4 and CCR5 were found to be strongly upregulated in CD8 T cells of patients with severe COVID‐19 infection, mainly presenting with pulmonary failure. As enhanced activation of the adaptive immune system is of utmost importance for clearing an infection, our data link clinical severity to altered chemokine receptor expression and stronger lymphocyte activation, and an increase in antibody secreting cells. Although patients did develop antibody titers independent of severity of the disease, functionality of those SARS‐CoV‐2‐specific antibodies needs to be further addressed in consecutive studies.

## Discussion

Our present study characterizes the different changes of the adaptive immune system as a consequence of COVID‐19, but it also suggests potential mechanisms associated with a more severe course of infection: higher T‐cell activation and T‐effector cell differentiation, increased exhaustion and reduction of senescent T cells. Furthermore, altered chemokine receptor expression could be linked to severe pulmonary symptoms. Our findings support other previous studies investigating the adaptive immune system during COVID‐19 [18, 26, 27]. However, the differentiation between mild and severe cases is crucial when it comes to explaining potential mechanisms of disease progression and deterioration of the patient. Predominantly in severe cases of COVID‐19, we observed strongly increased T‐effector cell differentiation with exhaustive phenotypes and disturbed T‐cell senescence, which can be linked to a loss of control in the T‐effector cell phase.

PD‐1 expression on T cells has not only been associated with T‐cell activation but also exhaustion in human HIV [28] or hepatitis B infection [29]. We found both CD4 and CD8 T cells presenting elevated PD1 and CTLA‐4 expression, molecules that have been associated with exhaustive phenotypes. Interestingly, despite expressing PD1 and CTLA‐4 at a higher percentage, T cells in patients with severe SARS‐CoV‐2 infection were not phenotypically exhaustive, but remained in the effector state and produced high amounts of effector molecules perforin, granzyme B, and IFN‐y. This raises the assumption that even in clinically severe cases functionality of T‐effector cells seemed to be maintained.

Besides activated and exhausted T cells, we also noted an increase in senescent T cells in mildly but not in severely diseased COVID‐19 patients compared to healthy individuals. Senescent T cells are characterized by secreting proinflammatory cytokines, such as IL‐1, IL‐6, and IL‐8, while they do not proliferate and expand anymore. An increase in senescent T cells has been associated with chronic viral infections and tumors [30, 31] and recent publications have also been associated with an increase in senescent T cells with COVID‐19 [26, 32]. We hypothesize, that T‐cell senescence might contribute to the control of the immune response during SARS‐CoV‐2 infection, a mechanism that could be impaired in severe cases of the disease. In terms of NK‐cell activation, we observed an increase in Ki67 positive NK cells with similar levels in mild and severe patients, pointing toward a robust NK‐cell activation which is in accordance with a publication by Maucourant and colleagues [33].

Besides T lymphocytes and NK cells, also B cells play a role in the control of COVID‐19, as patients with decreased B cells at diagnosis were reported to shed the virus for a longer period [14]. Consistent with a study by Mathew and colleagues, we found in our cohorts increased frequencies of plasmablasts and proliferating B cells [34]. When defining naïve B cells, memory B cells and plasmablasts, we relate to the expression of the CD27 and CD38 according to previous publications by Mathew et al. and Thevarajan et al. [13, 34]. B‐cell activation is characteristic in COVID‐19; interestingly, increased B‐cell activation leading to a lupus‐like phenotype has been reported in critically ill SARS‐CoV‐2‐infected patients [35]. Antibody responses are crucial for clearing infections. For SARS‐CoV‐2, it was reported that in a cohort of 285 patients, all patients tested positive for antiviral IgG after 19 days [36].

T‐cell‐mediated B‐cell activation is an initial step for antibody production, as B cells will develop into plasmablasts. In our cohort of patients infected with SARS‐CoV‐2, plasmablasts were significantly increased in both patients with mild and even more with severe COVID‐19 disease, compared to the cohort of healthy controls. Prior to B‐cell differentiation, “helper T cells” need to be activated in order to provide potent help to B cells in the germinal cells. The CD4^+^ CXCR5^+^ T_FH_ express PD1 and ICOS when being activated [37]. In our cohorts, we could note increasing numbers of ICOS^+^ PD1^+^ expressing circulating T_FH_ in the peripheral blood of patients with mild and severe SARS‐CoV‐2 infection, which is in line with a previous publication by Thevarajan and colleagues [13]. Other publications show a trend to higher numbers of CD4+ CXCR5^+^ PD1^+^ circulating T_FH_ in patients with mild COVID‐19 disease [27]. Furthermore, also the percentages of CD38 and HLA‐DR expressing, active circulating T_FH_ were significantly increased in COVID‐19 patients. This observation points toward a crucial involvement of T_FH_ activation for efficient SARS‐CoV‐2 specific antibody production. Interestingly, circulating PD1^+^ ICOS^+^ T_FH_ and plasmablasts are expanded in both mild and severe SARS‐CoV‐2 infection, consistent with previous reports showing that there is no correlation of antibody titers with clinical course of the disease [38].

Our data reveal increased T‐cell activation in patients with COVID‐19 including higher expression of PD1. Recently, a study by Sattler and colleagues show SARS‐CoV‐2‐specific CD4^+^ T cells to present higher levels of PD1. In patients failing to mount a specific T‐cell response, the mortality rate was much higher compared to those who achieved adequate SARS‐CoV‐2 T‐cell reactivity [39]. When looking at CD8 T cells, a study by Habel and colleagues shows SARS‐CoV‐2‐specific CD8 T cells to express PD1 only in patients with acute disease. Interestingly, also in healthy individuals SARS‐CoV‐2 reactive T‐cells could be detected [40, 41, 42], pointing toward a cross‐reactivity with other coronaviruses [15, 42]. The relevance of these preformed peptide‐specific T cells in fighting a SARS‐CoV‐2 infection, however, is not known, yet.

A potential limitation of our study is the different time points of blood draw ranging from a median of 5 days after onset of symptoms in mildly diseased patients up to a median of 11 days in patients with a severe course of disease. In our patients, clinical COVID‐19 disease was active, however, SARS‐CoV‐2 viral load was not present any more in eight patients (four severely diseased and four mildly diseased). All severely diseased patients have been treated in the ICU department at the time of blood collection due to complications from Covid‐19 (pneumonia, renal failure). However, time points of blood draw were heterogeneous, and usually later for the severely diseased patients, which is partially due to initial mild symptoms in these patients and secondary worsening. Nevertheless, SARS‐CoV‐2‐ specific T cells were shown to be present quite early after the onset of COVID‐19. They increased over time [43] and were still present in convalescent patients [44], demonstrating that later timepoints in severely diseased patients are not associated with a loss of T‐cell activation and functionality.

In all of our described patients with severe COVID‐19 disease, respiratory failure was the reason for admission to the ICU. We hypothesized that in those patients the local pulmonary immune response must be critically altered, presumably due to increased tracking of lymphoid cells to the lungs. Chemokines and chemokine receptors are pivotal in homeostatic and inflammatory migration of distinct immune cell types as they contribute significantly to the homing process of lymphocytes. In our cohorts, chemokine receptor expression on T cells was affected in COVID‐19 patients, especially in those experiencing a severe course of disease. Interestingly, CCR4 and CCR5 expression was upregulated in CD8 T cells, both chemokine receptors have already been associated with other viral infections. CCR5 expression has been linked to the entry of the HIV virus [45], however, CCR4 was reported to be induced upon HTLV‐1 viral infection [46]. Beyond this, CCR4 is important in T‐cell lung imprinting [25], as lung DC have been described to activate CCR4 expression on T cells. In the absence of CCR4, T cells do not traffic to the lungs which results in impaired antiviral responses against influenza virus. In this context, IL‐4‐expressing BALF T cells were shown to express the chemokine receptors CCR3 and CCR4 [47]. As CCR4 expression was highly increased both in CD4 and CD8 T cells in our severely diseased patients, we hypothesize that in severe cases of COVID‐19, increased numbers of CCR4 expressing T cells promote a stronger immune responses directly in the lungs. Presumably, CCR4 expressing T cells migrate to the pulmonary system, which leads to respiratory stress and even respiratory failure. Taken together, although the number of our analyzed patients is limited, our data are clear and representative and highlight the involvement of the adaptive immune system in the pathogenesis of SARS‐CoV‐2 infection with special regard to the clinical manifestation.

## Material and methods

### Human subjects

Sample collection and analysis were approved by the ethics committee of the Medical Center, University of Erlangen, Germany (protocol 118_20B and 174_20B). This study was conducted in accordance with the Declaration of Helsinki. We reviewed the patients` characteristics and medical history retrospectively from patients` medical charts. Clinical laboratory tests, including leukocyte and lymphocyte cell counts, CRP, and ferritin, were performed for each patient.

### Cell preparation and reagents

Peripheral blood of the indicated healthy donors or patients was collected into EDTA tubes (Cat. #02.1066.001, Sarstedt, Nuembrecht, Germany), PBMC were separated by density gradient centrifugation using Pancoll (PAN Biotech, Aidenbach, Germany) and stored in liquid nitrogen until further processing. Cells were thawed in RPMI‐1640 medium containing 10% human AB‐serum (both c.c.pro, Oberdorla, Germany), washed in PBS (Thermo Fisher Scientific, Waltham, USA), filtered with a 70 μm filter (BD Biosciences, Heidelberg, Germany), counted and aliquoted for staining. The following reagents were used for cell activation: PMA, Ionomycin (both Sigma‐Aldrich, Munich, Germany), and Golgi Stop (BD Biosciences).

### Flow cytometry

Cells were stained with 7AAD and anti‐human anti‐CD3 (clone UCHT1), anti‐CD4 (SK3), anti‐CD8 (RPA‐T8), anti‐CD14 (MoP9), anti‐CD19 (SJ25C1), anti‐CD27 (M‐T271), anti‐CD38 (HB7), anti‐CD40L (TRAP1), anti‐CTLA‐4 (BNI3), anti‐HLA‐DR (L243), anti‐interferon‐γ (IFN‐γ, B27), anti‐Ki‐67 (B56; BD Biosciences), anti‐CD25 (BC96), anti‐CD57 (HNK‐1), anti‐CD127 (A019D5), anti‐CCR4 (L291H4), anti‐CCR5 (J418F1), anti‐CCR6 (G034E3), anti‐CCR7(G043H7), anti‐CXCR3 (G025H7), anti‐CXCR5 (J252D4), anti‐Granzyme B (GB11), anti‐ICOS (C398.4A), anti‐IL‐4 (MP4‐25D2), anti‐PD1 (EH122H7), anti‐Perforin (B‐D48), and anti‐TNF (MAb11; Biolegend, San Diego, USA) monoclonal antibodies (mAbs). Intracellular Ki‐67, CTLA‐4, Granzyme B, and Perforin were stained with the Cytofix/Cytoperm kit (BD Biosciences), according to the manufacturer's protocol. Detection of intracellular cytokines was performed as described previously [48]. Briefly, cells were treated with PMA (2 μg/mL)/Ionomycin (1 μM) in presence of Golgi Stop for 4 h, stained with antibodies against surface proteins, washed, fixed, and permeabilized with Cytofix/Cytoperm Kit, and stained with antibodies against IFN‐γ, IL‐4, TNF, and CD40L. Lymphocytes were determined by FSC‐A/SSC‐A, doublets were excluded by FSC‐A/FSC‐H, dead cells and monocytes were excluded by 7AAD and CD14, and cells were gated by the indicated mAbs. Flow cytometry data were acquired on a LSR Fortessa (BD Biosciences) and cells were analyzed for indicated mAbs with FlowJo software version 10 (TreeStar, Ashland, Oregon, USA) and Kaluza software v2.1 (Beckmann Coulter, Krefeld, Germany). Gating strategies for lymphocyte populations and CD4 T‐cell subsets are shown in Supporting information Figs. S2 and S6. Flow cytometry was performed according to the published guidelines by Cossarizza et al. [49].

### Chemiluminescent immunoassay (CLIA)

Commercially available magnetic bead‐based chemiluminescent immunoassay (CLIA) for the detection of SARS‐CoV2‐specific IgG (N‐ and S‐specific, Shenzhen Yhlo Biotech, iFlash‐SARS‐CoV‐2, Cat #C86095G) were performed on a fully automated iFlash Immunoassay Analyzer (Shenzhen Yhlo Biotech). The assays were performed according to the manufacturer's protocols. The levels of specific IgG were given as arbitrary units (AU/mL) and the cut‐off value for a positive test was 10 AU/mL.

### Multiplex immunoassay

For detection of cytokines in the serum of the patients, we used a multiplexed immunoassay from Mesoscale Discovery (MSD), basing on conventional sandwich ELISA technology with electrochemiluminescence signal detection. The cytokines and chemokines were detected with a precoated V‐PLEX Human Biomarker 46‐Plex array. The assays were performed according to manufacturer's protocol with incubation of the diluted samples and standards overnight at 4°C in the dark. The electrochemiluminescence signal was detected on a MESO QuickPlex SQ 120 plate reader (MSD) and analyzed with Discovery Workbench Software (version 4.0, MSD). The concentration of the samples is calculated based on the four‐parameter logistic fitting model, which was generated with the respective standards (the concentration can be determined according to the certificate of analysis provided by the manufacturer).

### Statistical analyses

Data were analyzed with Graphpad Prism 8.3.0 (GraphPad San Diego, California, USA). Results were compared using nonparametric Mann—Whitney U test. Spearman`s rank test was used for correlation analysis. A *p*‐value of <0.05 was considered significant.

## Author contributions

S.S., A.N.K., K.Ü., T.W., A.M., A.E.K., and S.V. designed the research; A.E.K., G.K., and M.V. repeatedly referred patients; S.S., N.E., P.K., L.M., P.L., H.B., C.M, G.L‐G., M.T., L.H., B.F., and S.V. performed experiments; S.S., A.N.K., M.A., N.E., H.B., M.N., A.E.K., and S.V. analyzed and interpreted data; S.S., A.N.K., A.M., A.E.K., and S.V. wrote the manuscript; and all of the authors edited the manuscript.

## Conflict of interest

All authors declare no commercial or financial conflict of interest.

### Peer review

The peer review history for this article is available at https://publons.com/publon/10.1002/eji.202049135.

**Abbreviations**
AU: arbitrary units
CLIA: chemiluminescent immunoassay
CM: central memory
COVID‐19: coronavirus disease 2019
CRP: C‐reactive protein
CTLs: cytotoxic T lymphocytes
ICU: intensive care unit
mAbs: monoclonal antibodies
MSD: mesoscale discovery
PD‐1: programmed cell death receptor 1
SARS‐CoV‐2: severe acute respiratory syndrome coronavirus 2
T_FH_: T‐follicular helper cells

## Supporting information

Supporting Material

## Data Availability

The data that support the findings of this study are available on request from the corresponding author. The data are not publicly available due to privacy or ethical restrictions.
