# Supplementary material for: Upregulation of CCR4 in activated CD8+ T cells indicates enhanced lung homing in patients with severe acute SARS‐CoV‐2 infection
Source: Eur J Immunol. 2021 Apr 19;51(6):1436–48. doi: 10.1002/eji.202049135 (PMC8250120; doi:10.1002/eji.202049135)
Supplement: Supplementary file 1 — Supporting Material [file EJI-51--s001.pdf]

Supplemental Fig. 1

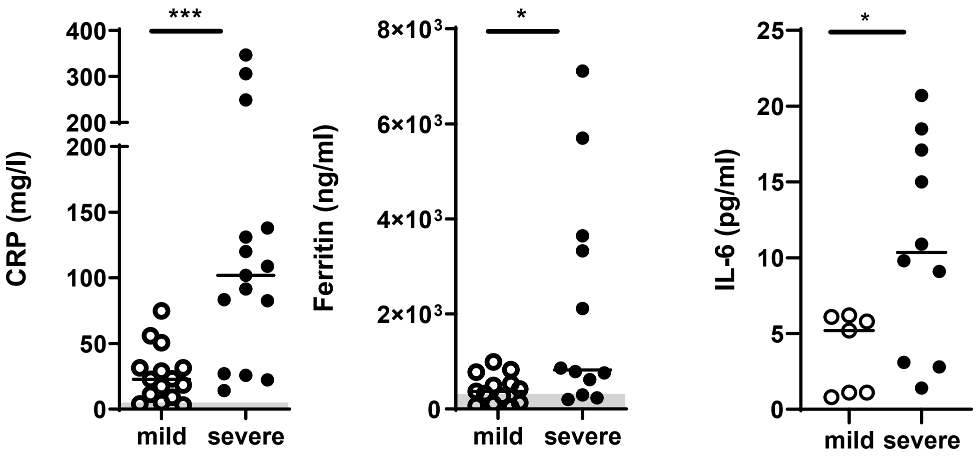

Supplemental Figure 1. CRP, ferritin and IL-6 levels in patients with mild (open circle) versus severe (black circle) COVID-19 disease are shown. Healthy donor range is indicated in grey. Bars represent median, *p<.05, and ***p<.001 (Mann-Whitney-U test). Each symbol represent an individual subject (CRP: mild: n=15, severe: n=15; ferritin: mild: n=13 and severe: n=12). For IL-6 levels, data were available of 7 patients with mild and 10 patients with severe SARS-CoV-2 infection.

Supplemental Fig. 2

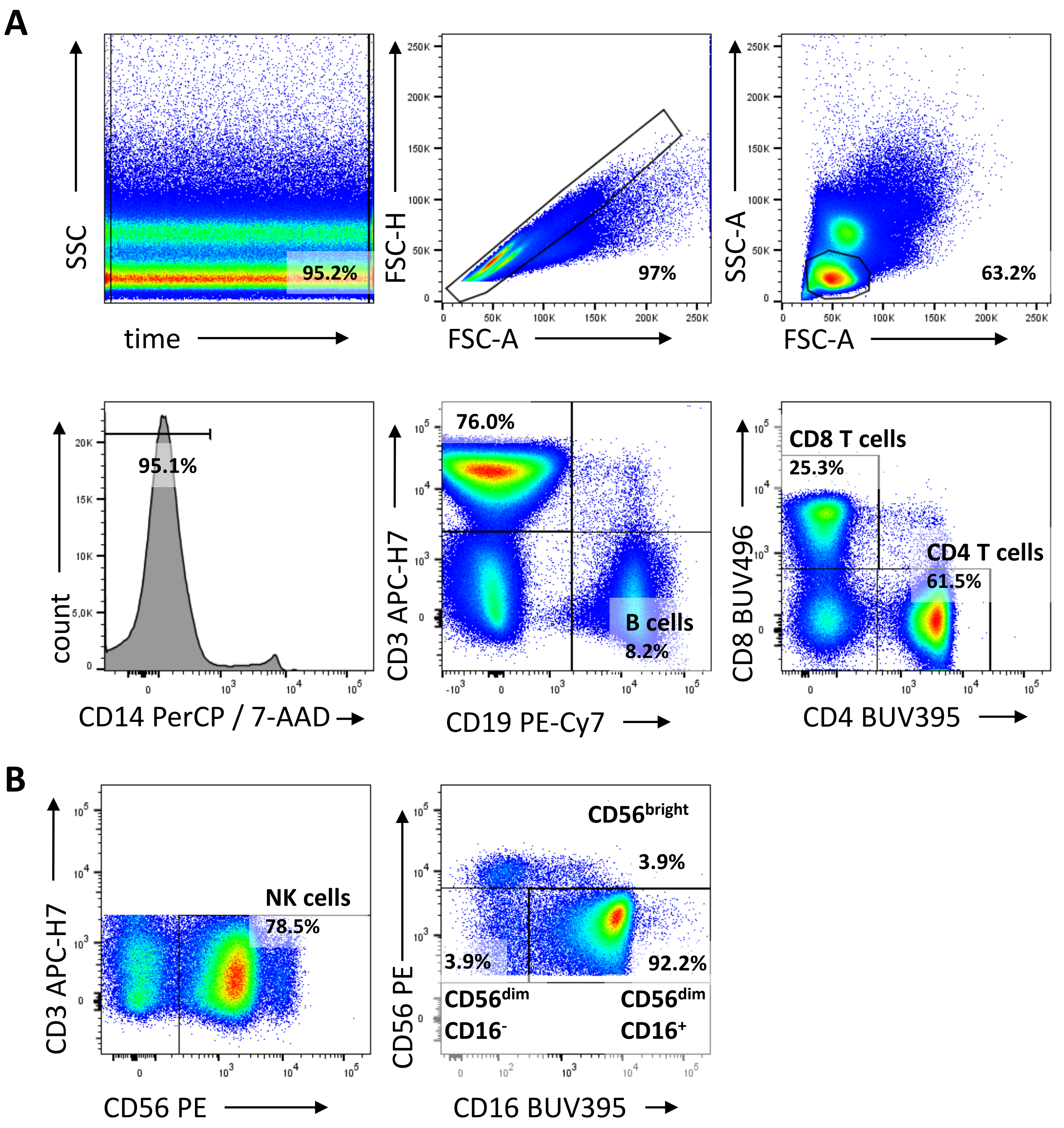

Supplemental Figure 2. Gating strategy of flow cytometry: Time parameter was used to monitor instrument stability, doublets were excluded by FSC-H/FSC-A, cells were determined by FSC-A/SSC-A, remaining monocytes and dead cells and were excluded by CD14 and 7-AAD. (A) B cells were determined by CD19^{+}/CD3^{-} gate, T cells were gated by CD3^{+} and CD19^{-} and further subdivided in CD4^{+} T cells and CD8^{+} T cells. (B) NK cells were gated as CD3^{-}/CD19^{-}/CD56^{+} and further subdivided in CD56^{bright}, CD56^{dim}/CD16^{+} and CD56^{dim}/CD16^{-} NK cells.

Supplemental Fig. 3

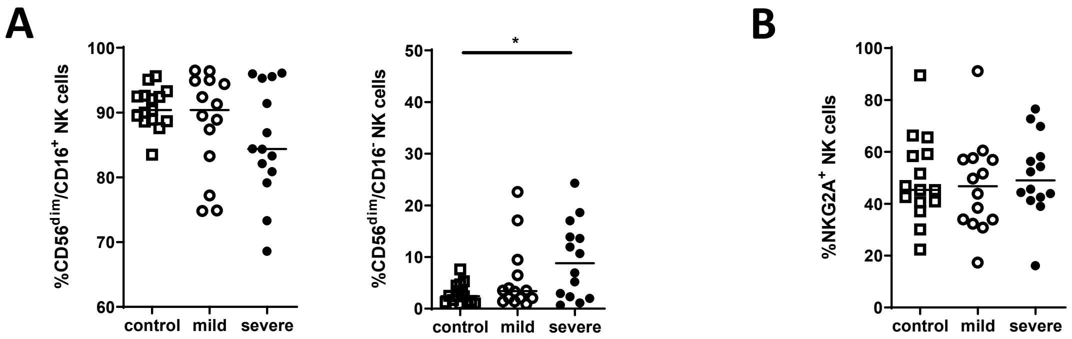

Supplemental Figure 3. NK cell expression profile in patients with SARS-CoV2 infection and healthy controls. (A) NK cells were gated for CD56^{dim} and CD16 expression. (B) NKG2A expression on NK cells were analyzed by flow cytometry. Graphs exhibit cumulative data of all COVID-19 patients (mild: open circle; severe: black circle) and healthy controls (open squares), each symbol represent an individual subject. Bars represent median, significance within these cohorts is calculated using Mann-Whitney-U test with *p<.05.

Supplemental Fig. 4

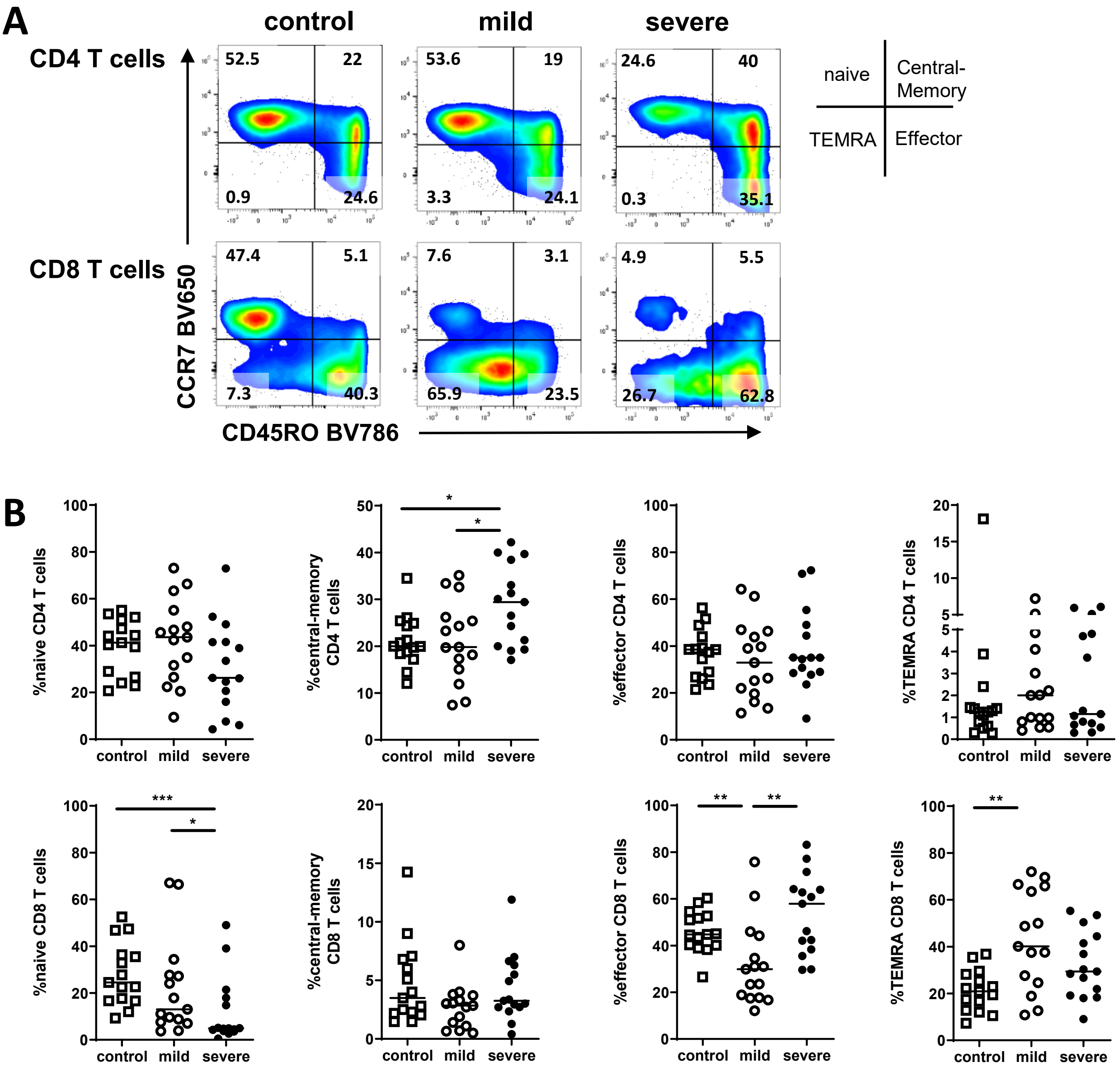

Supplemental Figure 4. T cell differentiation in patients with SARS-CoV2 infection and healthy controls. CD4 T cells and CD8 T cells were analyzed for CCR7 and CD45RO expression by flow cytometry. Naïve T cells were gated as $CCR7^{+}/CD45RO^{-}$, central-memory cells were gated as $CCR7^{+}/CD45RO^{+}$, effector cells were gated as $CCR7^{-}/CD45RO^{+}$, and TEMRA cells were gated as $CCR7^{-}/CD45RO^{-}$. (A) Representative density plots are shown. (B) Graphs exhibit cumulative data of all COVID-19 patients (mild: open circle; severe: black circle) and healthy controls (open squares), each symbol represent an individual subject. Bars represent median, significance within these cohorts is calculated using Mann-Whitney-U test with *p<.05, **p<.01 and ***p<.001.

Supplemental Fig. 5

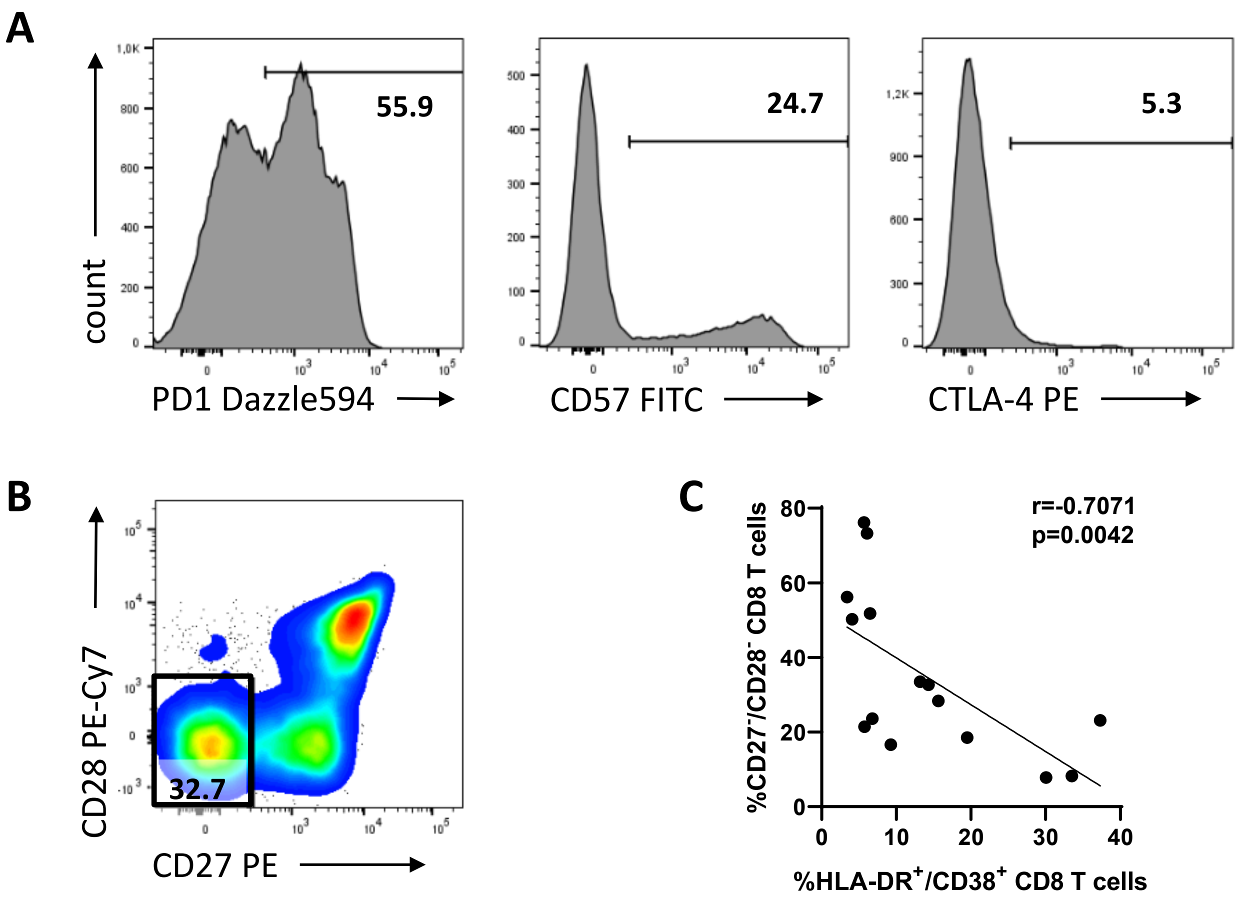

Supplemental Figure 5. Expression of PD1, CD57, CTLA-4, CD27, and CD28 were determined in CD8 T cells of a COVID-19 patient. (A) Representative histograms depicting levels of receptor expression are shown, CTLA-4 expression was stained intracellularly. (B) Density plot shows frequency of $CD27^-/CD28^-$ CD8 T cells.(C) Correlation of percentages of $CD27^-/CD28^-$ and $HLA\text{-}DR^+/CD38^+$ cells among all CD8 T-cells from COVID-19 patients with severe disease (n=15) is shown. r=-0.7071, **p<.01 (Spearman's test).

Supplemental Fig. 6

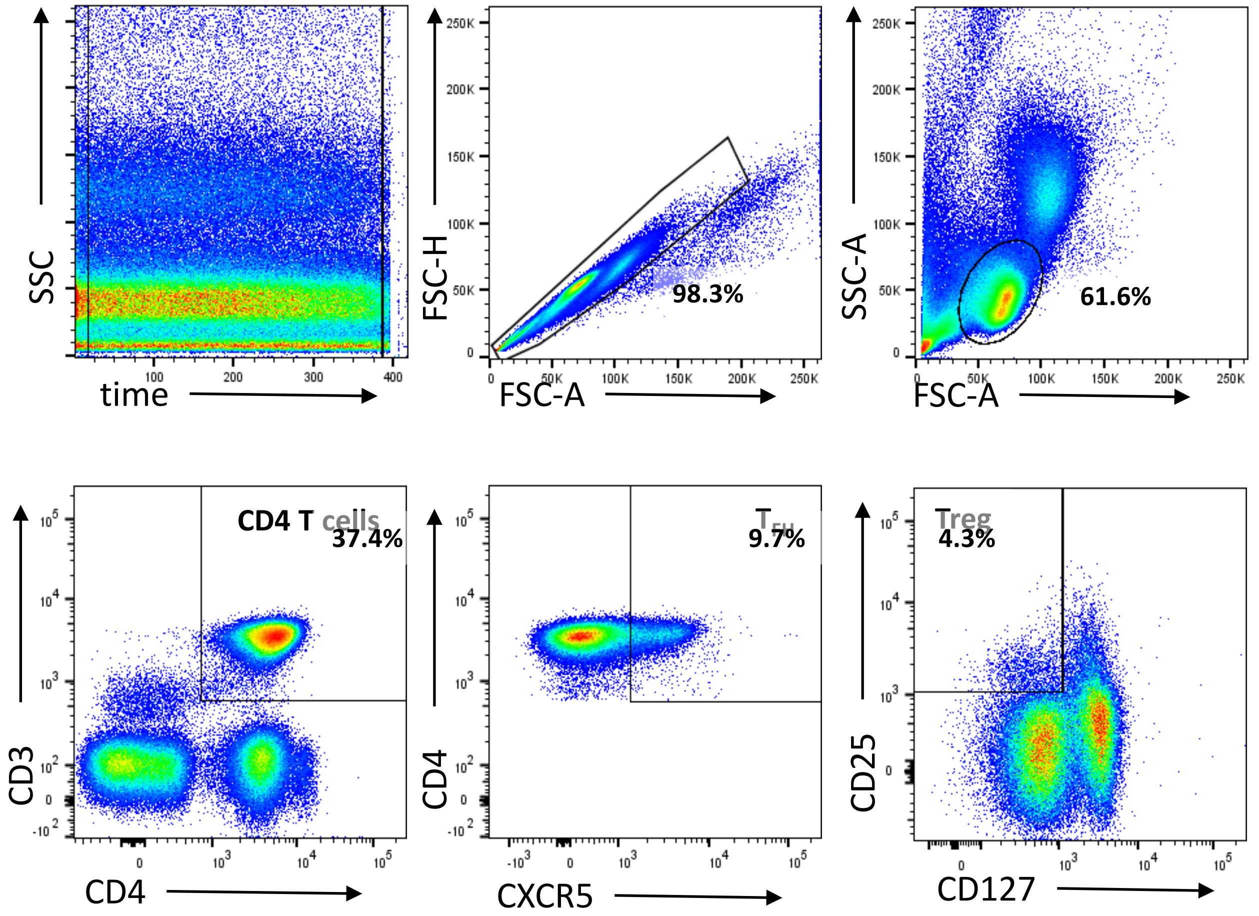

Supplemental Figure 6. Gating strategy for assessment of circulating T_{FH} and Treg cells. Time parameter was used to monitor instrument stability, doublets were excluded by FSC-H/FSC-A, lymphocytes were determined by FSC-A/SSC-A, CD4 T helper cells were gated by $CD3^+$ and $CD4^+$ and further subdivided in $CXCR5^+$ T_{FH} and $CD25^+/CD127^-$ Treg cells.

Supplemental Fig. 7

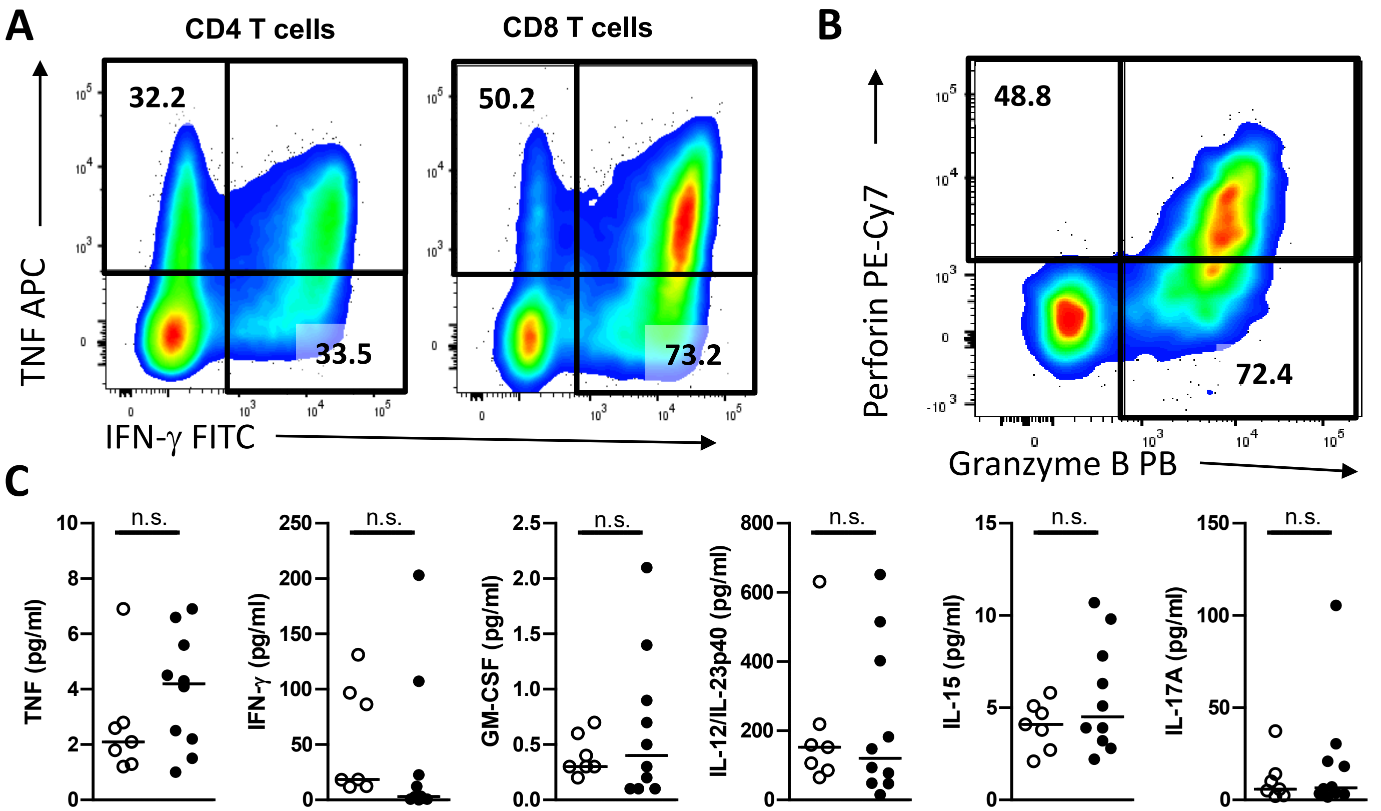

Supplemental Figure 7: T cell functionality in COVID-19 patients. (A) PBMC of a COVID-19 patient were stimulated with PMA/ionomycin in the presence of Golgi Stop. Expression of TNF and IFN-γ was determined in CD4 and CD8 T cells by intracellular flow cytometry staining. (B) CD8 T cells of a COVID-19 patient were analyzed for intracellular expression of the cytotoxic molecules Perforin and Granzyme-B. (C) Systemic levels of inflammatory cytokines in patients with SARS-CoV-2 infection. Serum levels of indicated cytokines in patients with mild (open circle) versus severe (black circle) COVID-19 disease are shown. Bars represent median, significance within these cohorts is calculated using Mann-Whitney-U test (n.s. not significant). Data are available of 7 patients with mild and 10 patients with severe SARS-CoV-2 infection.

Supplemental Fig. 8

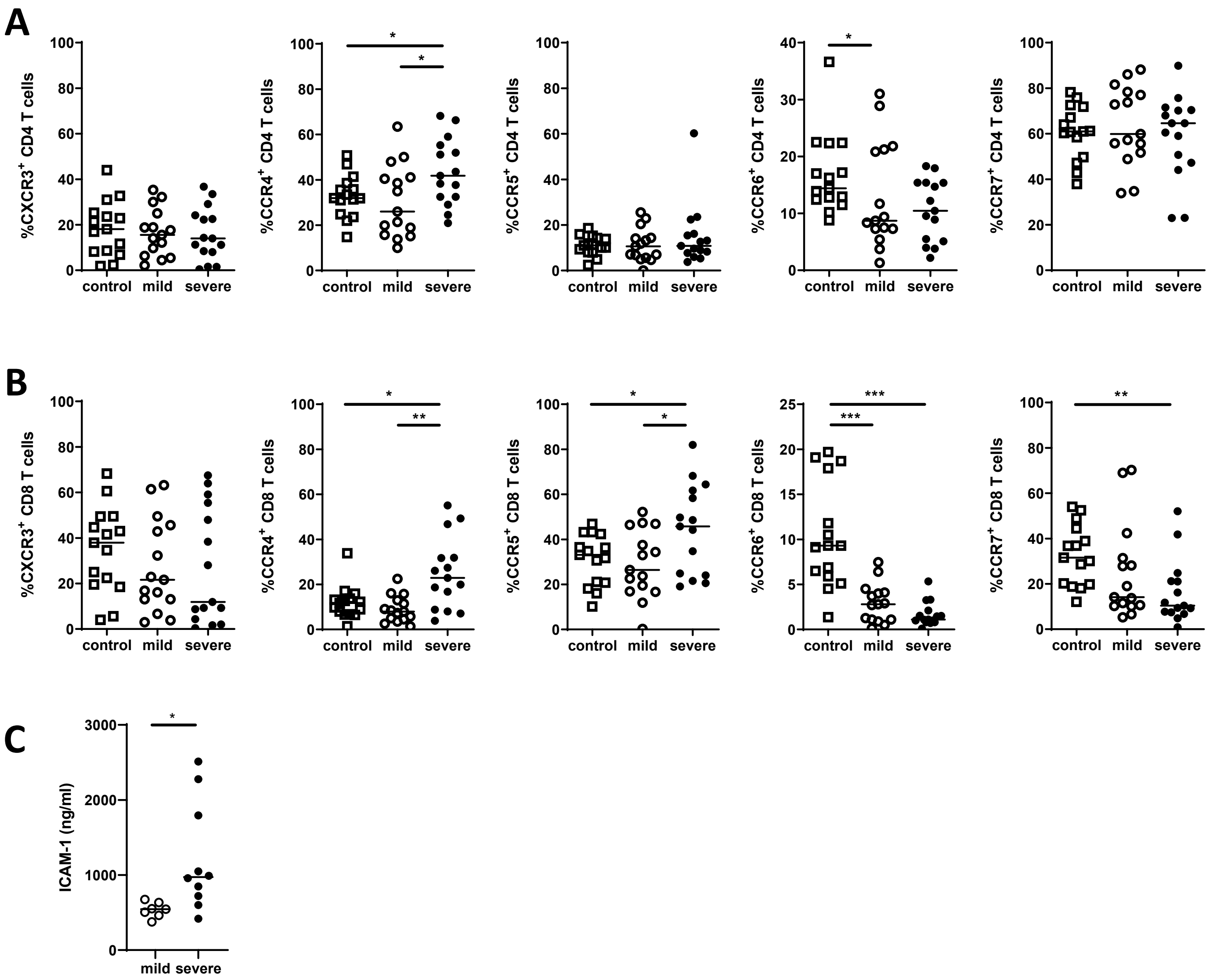

Supplemental Figure 8. Chemokine receptor expression profile of T cells from patients with SARS-CoV2 infection. Expression of CXCR3, CCR4, CCR5, CCR6, and CCR7 on CD4 T cells (A) and CD8 T cells (B) was determined by flow cytometry. Graph exhibit percentages of receptor positive cells, each symbol represent an individual subject. Data from healthy individuals (open square, n=15) and COVID-19 patients with mild (open circle, n=15) and severe disease (black circle, n=15) is shown. Bars represent median, significance within these cohorts is calculated using Mann-Whitney-U test with *p<.05, **p<.01 and ***p<.001. (C) Serum levels of ICAM-1 in patients with mild (open circle) versus severe (black circle) COVID-19 disease are shown. Bars represent median, *p<.05 (Mann-Whitney-U test). Data were available of 7 patients with mild and 10 patients with severe SARS-CoV-2 infection.

ID	Age	Sex	Severity (NIH scale)	Total days on ICU	Mechanical ventilation	Outcome	Days after onset of symptoms	Viral load (CT values)	WBC (/µl)	ALC (/µl)	CRP (mg/l)	Ferritin (ng/ml)	SARS-CoV-2-IgG (AU/ml)	Comorbidities	ACE-I
0002	65	m	5	0	no	alive	3	25.7	5150	600	31,5	322	0	TIA, meningeoma, AHT	no
0010	65	f	5	0	no	alive	5	25.6	3760	1100	3,2	134	0	OSAS, obesitas, AHT	no
0109	96	f	5	0	no	alive	7	neg (d4)	6700	2280	4,9	370	77	AHT, WPW syndrome	no
0050	78	m	5	0	no	alive	1	19.7	4550	1200	18,4	45	1	CKD stage 5, AFib, AHT, hemiparesis after ICB, A. vertebralis-syndrome, s/p prostate cancer	no
0051	57	f	5	0	no	alive	13	36.9	4260	1200	22,8	536	41	*Simultaneously: lobar pneumonia*	no
0052	44	m	5	0	no	alive	15	22.9	4320	950	50,7	777	0	none	no
0054	92	f	4	0	no	alive	5	30.7	6960	1100	74,9	275	63	AHT, ARF, heart failure, s/p stroke, asthma	no
0067	68	m	5	0	no	alive	14	33.7	4620	600	55,9	828	11	DLBCL IIIB, SA-block with pacemaker, diabetes mellitus type 2	no
0077	53	m	5	0	no	alive	4	28.6	5930	3200	31.4	991	1	Immuneneutropenia, AHT, hypercholesterolemia	no
0081	82	m	5	0	no	alive	4	37.1	6610	1650	29,1	419	21	AHT, diabetes mellitus type 2, hypercholesterolemia, hypothyroidism	yes
0087	34	m	5	0	no	alive	7	20.2	3230	880	9,1	494	0	none	no
0090	52	d	4	0	no	alive	14	neg. (14d)	5610	1800	11,4	n.d.	54	s/p PE, Klinefelter-Syndrome, s/p sex change, depression, hypothyroidism	no
0294	71	f	6	0	no	alive	no sympt.	neg. (2d)	8100	2745	3,9	n.d.	70	Lung cancer, paraneoplatic neuropathy and encephalitis, CHD, AHT	yes
0378	18	f	5	0	no	alive	1	neg. (1d)	16380	600	23,2	108	9	none	no
0379	23	f	5	0	no	alive	5	23.5	4510	1200	17,4	71	4	none	no
0091	74	m	2	21	yes	alive	33	neg. (5d)	16460	3200	82,5	n.d.	70	CHD, heart failure, obesitas, diabetes mellitus type 2, mitral valve replacement, AHT, AFib, prostate hyperplasia	no
0106	88	f	1	17	no (DNI)	dead	20	31.4	40830	2040	25,8	236	76	MPN, AFib, heart failure, AHT, aortic valve replacement	yes
0130	63	m	2	68	yes, incl. ECMO	alive	60	neg. (12d)	8090	1800	91,5	3330	56	AHT, obesitas per magna, tricuspid insufficiency	no
0131	75	m	2	45	yes	alive	35	31.7	6800	2900	102	5699	3	CHD, CLL, PAD, AFib	yes
0132	64	f	2	44	yes	alive	7	19.4	14380	800	138	786	44	MGUS, AHT	no
0133	73	m	2	82	yes, incl. ECMO	alive	41	neg.(21d)	13000	8700	22,4	7109	92	Glaucoma, HIT type II	no

0230	77	f	1	12	no (DNI)	dead	37	26.1	14220	1200	131	758	72	Diabetes mellitus type II, dementia, ARF, peripheral neuropathy, AHT	yes
0265	69	m	2	67	yes, incl. ECMO	alive	67	neg. (18d)	7420	1470	14,2	2117	83	AHT, prostate hyperplasia	yes
0035	85	f	1	0	no (DNI)	dead	2	19.7	5340	1400	120	862	0	Dementia, AHT, depression, ARF, obesitas	no
0057	37	m	2	30	yes	alive	3	26.7	5010	1500	26,9	203	1	Paraplegia after ICB	no
0280	66	f	1	10	yes	dead	2	19.4	15340	1100	347	621	n.d.	Hypothyroidism, metabolic syndrome	unknown
0303	58	f	1	3	yes	dead	11	34.8	2940	970	306	3644	80	AML, AFib, s/p Mamma-CA	no
0383	62	f	2	>20*	yes	alive*	10	24.3	10580	1500	249	298	38	Cervical malformation	no
0384	70	m	2	>13*	yes	alive*	5	20	5350	700	83,3	n.d.	2	Tonsille-CA, CHD, AHT, Diabetes mellitus type II	no
0385	62	f	3	7	no	alive	10	37.6	10570	4100	108,8	n.d.	40	AHT, hypothyroidism, asthma	yes

Supplemental Table 1: Characteristics of patients with mild and severe COVID-19 infection. The following information was drawn retrospectively from electronic, medical charts: Age, gender, severity of COVID-19 according to NIH scale, length of ICU treatment, outcome, time from onset of disease symptoms until the blood draw, CT values of viral load at the time of blood sample collection, laboratory values as white blood count (WBC), ALC (absolute lymphocyte count), CRP, ferritin, SARS-COV-2 specific IgG levels, comorbidities, and medication history (ACE inhibitors). If viral load was already negative at the time point of blood draw, the duration since the last positive swab is indicated in brackets.
*: still under treatment

Abbr.: AFib: atrial fibrillation; AHT: arterial hypertension; ARF: acute renal failure; CHD: coronary heart disease; CKD: chronic kidney disease; CLL: chronic lymphocytic leukemia; DLBCL: diffuse large B-cell lymphoma; DNI: do not intubate, HIT: heparin-induced thrombocytopenia; ICB: intracranial bleeding; MGUS: monoclonal gammopathy of unknown significance; MPN: myeloproliferative neoplasia; OSAS: obstructive sleep apnea; PAD: peripheral artery disease; TIA: transient ischemic attack;
